# Low-Density Parity-Check Decoding Algorithm Based on Symmetric Alternating Direction Method of Multipliers

**DOI:** 10.3390/e27040404

**Published:** 2025-04-09

**Authors:** Ji Zhang, Anmin Chen, Ying Zhang, Baofeng Ji, Huaan Li, Hengzhou Xu

**Affiliations:** 1 School of Mathematics and Statistics, Henan University of Science and Technology, Luoyang 471000, China; 2 Intelligent System Science and Technology Innovation Center, Longmen Laboratory, Luoyang 471000, China; 3 College of Physics and Telecommunication Engineering, Zhoukou Normal University, Zhoukou 466001, China; 4 School of Computer, Henan University of Engineering, Zhengzhou 451191, China

**Keywords:** alternating direction method of multipliers, symmetric ADMM, low-density parity-check codes, penalized decoding

## Abstract

The Alternating Direction Method of Multipliers (ADMM) has proven to be an efficient approach for implementing linear programming (LP) decoding of low-density parity-check (LDPC) codes. By introducing penalty terms into the LP decoding model’s objective function, ADMM-based variable node penalized decoding effectively mitigates non-integral solutions, thereby improving frame error rate (FER) performance, especially in the low signal-to-noise ratio (SNR) region. In this paper, we leverage the ADMM framework to derive explicit iterative steps for solving the LP decoding problem for LDPC codes with penalty functions. To further enhance decoding efficiency and accuracy, We propose an LDPC code decoding algorithm based on the symmetric ADMM (S-ADMM). We also establish some contraction properties satisfied by the iterative sequence of the algorithm. Through simulation experiments, we evaluate the proposed S-ADMM decoder using three standard LDPC codes and three representative fifth-generation (5G) codes. The results show that the S-ADMM decoder consistently outperforms conventional ADMM penalized decoders, offering significant improvements in decoding performance.

## 1. Introduction

The linear programming (LP) decoder, initially proposed by Feldman et al. [1,2,3], serves as an approximation to maximum-likelihood (ML) decoding and is widely recognized as a key technique for decoding binary linear codes. Despite its theoretical strength, traditional LP decoding, when solved using generic solvers, is computationally demanding and can lead to considerable delays. This makes it less practical for real-time applications, where decoding speed is critical. To overcome this limitation, Barman et al. [4] introduced a more efficient and tractable decoding framework by applying the Alternating Direction Method of Multipliers (ADMM) [5]. This approach significantly improves computational efficiency while retaining the benefits of LP decoding. The ADMM-based LP decoder offers a decoding performance that is comparable to that of belief-propagation (BP) decoders [6], making it a more viable option for practical decoding in real-world systems. Additionally, the ADMM-LP decoder provides a scalable and faster alternative, especially in scenarios where low latency and high throughput are essential.

Currently, research on ADMM decoding can be broadly classified into several key areas. One direction focuses on accelerating the decoding process. Several studies aim to reduce the complexity of ADMM-LP decoding [7,8,9,10,11,12]. Wei et al. proposed an iterative projection algorithm that has linear complexity in the worst case in [11]. Xia et al. proposed a line segment projection algorithm [12] that does not require sorting or iterative operations, and conducted further research to improve this algorithm. The line segment projection algorithm is an approximate projection algorithm with low complexity, which saves more projection time compared to cut search algorithms. However, its decoding performance is somewhat inferior to that of more precise projection algorithms. In addition to improving projection techniques, other studies have taken different approaches to enhance the efficiency of ADMM decoding. Bai et al. proposed a check polytope-free ADMM framework in [13], which bypasses the need for complex check polytope computations, further reducing the overall computational burden. Yang et al. demonstrated in [14] that linear complexity offered a more scalable solution to decoding as the code length increases. In [15], Xia et al. introduced an efficient hybrid projection algorithm based on the even vertex projection algorithm (EVA), which combines different projection techniques to minimize decoding time while maintaining performance. In [16], the author proposed a sparse affine projection algorithm that projects vectors onto the affine shell of vertices, reducing the complexity of each iteration. Hierarchical ADMM decoding [17] and node scheduling [18] have successfully reduced the required decoding iterations by changing the update strategy during the decoding process.

Another critical feature of LDPC codes is their decoding performance. One of the challenges in LDPC decoding arises from the fact that the LP model relaxes the binary constraints typically imposed on the variables, allowing them to take continuous values instead of being strictly binary. As a result, the solution can sometimes be fractional, leading to suboptimal results. To mitigate this issue, researchers have explored various techniques to refine the decoding process and ensure that the decoder outputs integral solutions. In [6], the authors introduced a penalty term. This penalty term is designed to assign smaller values to integral points, making them more favorable compared to fractional solutions. By doing so, the penalty encourages the algorithm to converge toward integer solutions that correspond to valid codewords, thereby improving the decoding accuracy and performance. Building on this idea, Jiao et al. [19] assigned distinct penalty parameters based on the node degree; the method allows for more flexibility in how penalties are applied, leading to better decoding performance, particularly in the context of irregular LDPC code. This approach helps to balance the penalties across the network, further enhancing the likelihood of the decoder outputting a valid codeword. This characteristic enhances the reliability of the decoding process, ensuring that the solutions converge to valid codewords more efficiently. The authors also explored and compared a variety of penalty functions to further optimize decoding performance, considering different strategies for balancing the penalties between valid and invalid solutions. Wang et al. [20] improved performance by allowing for a more nuanced penalization of invalid solutions, thus enhancing the likelihood of finding the correct codeword. In another study, ref. [21] introduced an innovative penalty term that focused on penalizing check nodes rather than variable nodes. This approach targeted the constraints more directly, leading to a more effective decoding strategy, particularly for certain types of error patterns.

Additionally, some researchers have proposed new constraints that perform functions similar to penalty terms but with distinct mathematical formulations. For example, Wu et al. [22] introduced LP-box constraints, which impose bounds on the values that the variables can take, providing a new way of controlling the solution space and improving decoding efficiency. In another work [23], the authors further refined the solution space and enhanced the accuracy of the decoding process. Moreover, ref. [24] proposed a restartable ADMM decoding framework, which allows for periodic resets of the algorithm to avoid local minima and improve overall decoding performance.

Due to the non-convexity of the objective function, the Lagrange multipliers need to be updated in each iteration to handle constraints by applying the ADMM penalty decoding. The convergence speed of the ADMM is slow, resulting in a significant increase in the number of iterations. Especially when the signal-to-noise ratio (SNR) is low, the decoding performance will be worse, which cannot meet the low latency requirements of 5G communication scenarios.

In this paper, unlike ADMM non-convex quadratic penalized decoders, we propose an LDPC code decoding algorithm based on the symmetric ADMM (S-ADMM). The key difference lies in updating the multiplier immediately after minimizing the first variable, followed by minimizing the second variable and updating the multiplier again. Therefore, theoretically, this approach should yield superior numerical results. Additionally, to ensure global convergence of the iterative sequence, we introduce a contraction factor in the multiplier update. There is an intermediate equation for updating the Lagrange multipliers. We refer to the proposed decoding method as the S-ADMM penalized decoder. In this approach, the penalty terms applied to all valid codewords are uniform and smaller compared to those assigned to invalid solutions. The penalty for valid codewords is carefully chosen to encourage solutions that satisfy the desired decoding conditions. On the other hand, invalid solutions, such as binary solutions that fail to meet all parity-check equations or non-binary solutions are assigned significantly larger penalty terms. This differential penalization ensures that invalid solutions are heavily discouraged, effectively reducing their likelihood of attaining the minimum objective function value. Consequently, the decoder is more likely to converge to a valid codeword, as the penalties imposed on invalid solutions make them increasingly unfavorable in the optimization process.

The main contributions of this paper are summarized as follows:

- We adopt a split optimization strategy to speed up the convergence for the ADMM decoding algorithm in handling non-convex quadratic models. By ensuring the relative independence and stability of each update, we solve the problem of unstable convergence of the ADMM in non-convex problems;
- We propose the S-ADMM decoding algorithm based on penalty terms and derive the algorithm process for the S-ADMM decoding model;
- We establish some contraction properties satisfied by the iterative sequence of the S-ADMM algorithm;
- Simulations demonstrate that the S-ADMM decoding algorithm outperforms the ADMM penalized decoders.

The remainder of this paper is organized as follows. In Section 2, we provide a comprehensive overview of the LP decoding model and introduce the ADMM penalized decoding approach, discussing its foundational principles and applications in decoding. Section 3 is dedicated to the presentation of the S-ADMM decoding model. We outline the specific formulation of the ADMM algorithm tailored to solve this model, and describe the overall decoding framework that integrates the proposed method. In Section 4, we thoroughly analyze the contraction properties of the S-ADMM decoder. Section 5 presents extensive simulation results, where we compare the performance of the S-ADMM decoder against existing methods, demonstrating its superior decoding efficiency and accuracy in various scenarios. Section 6 offers concluding remarks and summarizes the contributions of this work.

## 2. Preliminaries

Assume we are given an LDPC code of length *n* and dimension *k* specified by its (n−k)×n parity-check matrix *H*. Use I={1,2,…,n} and J={1,2,…,m} to represent the set of all variable nodes and check nodes, where m=n−k. $N_{v}\left(i\right)$ is the set of all the check nodes connected to variable node $v_{i}$, $d_{v_{i}}$ represents the degree of $v_{i}$, and similarly, $N_{c}\left(j\right)$ is the set of all variable nodes connected to check node $c_{j}$, and $d_{c_{j}}$ represents the degree of $c_{j}$. For regular LDPC codes, all check nodes have the same degree $d_{c_{j}}$, and all variable nodes have the same degree $d_{v_{i}}$.

### 2.1. LDPC Decoding Algorithm Based on ADMM-LP

Assuming that vector x = $\left(x_{1},x_{2},\ldots ,x_{n}\right)$ is the codeword to be transmitted, x∈C, C is the code set, and the received codeword is $w\in (w_{1},w_{2},\ldots ,w_{n})$. By using the maximum-likelihood decoding algorithm, it can be transformed into an optimization problem.

(1) $$\begin{array}{cc} \min_{x}\; & \gamma^{T}x,\\ s.\;t.\;T_{j}x & \in {PP}_{d_{c_{j}}},j\in J, \end{array}$$

where γ represents the logarithmic likelihood ratio (LLR) vector of the codeword after passing through the channel, and its *i*-th entry is $\gamma_{i}=\log\frac{E\left(w_{i}|x_{i}=0\right)}{E\left(w_{i}|x_{i}=1\right)}$. Each row of the check matrix corresponds to a check node, which is a check equation. Then, the selection matrix $T_{j}$ can be used to represent the variable node *v* connected to the check node *c*, with a size of $d_{c_{j}}\times n$. For example, for a certain row of the check matrix $h_{j}=\left(1,0,0,1,1,0,0,0,1\right)$, then $T_{j}$ will be

$$T_{j}=\left(\begin{array}{ccccccccc} 1 & 0 & 0 & 0 & 0 & 0 & 0 & 0 & 0\\ 0 & 0 & 0 & 1 & 0 & 0 & 0 & 0 & 0\\ 0 & 0 & 0 & 0 & 1 & 0 & 0 & 0 & 0\\ 0 & 0 & 0 & 0 & 0 & 0 & 0 & 0 & 1 \end{array}\right).$$

$P_{d_{c_{j}}}$ represents the parity polytope corresponding to the *j*-th parity node. So, we transformed Equation (1) into the solving framework of the ADMM.

(2) $$\begin{array}{cc} \min_{x}\; & \gamma^{T}x,\\ s.\;t.\;T_{j}x & =z_{j},\;z_{j}\in {PP}_{d_{c_{j}}},j\in J, \end{array}$$

$z=(z_{1},z_{2},\ldots ,z_{m})$ is the auxiliary variable. The augmented Lagrangian function of the constrained optimization problem (2) is

(3) $$L_{\beta }\left(x,z,\lambda \right)=\gamma^{T}x+\sum_{j}\lambda_{j}^{T}\left(T_{j}x-z_{j}\right)+\frac{\beta }{2}\sum_{j}{∥T_{j}x-z_{j}∥}_{2}^{2},$$

where $\lambda =(\lambda_{1},\lambda_{2},\ldots ,\lambda_{m})$ is Lagrangian multiplier, and β is the penalty parameter.

(4) $$\left\{\begin{array}{c} x^{k+1}=\underset{x}{\arg\min}\;\left\{L_{\beta }\left(x,z^{k},\lambda_{j}^{k}\right)\right\},\\ z_{j}^{k+1}=\underset{z}{\arg\min}\;\left\{L_{\beta }\left(x^{k+1},z_{j},\lambda_{j}^{k}\right)\right\},\\ \lambda_{j}^{k+1}=\lambda_{j}^{k}+\beta \left(T_{j}x^{k+1}-z_{j}^{k+1}\right). \end{array}\right.$$

The update rules for each iteration are shown in Equations (5a)–(5c).

(5a) $$\begin{array}{cc} x_{i}^{k+1} & =\prod_{\left[0,1\right]}\left(\frac{1}{d_{v_{i}}}\left(\sum_{j\in N_{v}\left(i\right)}{\left((z_{j}^{\left(i\right)}\right)}^{k}-\frac{{\left(\lambda_{j}^{\left(i\right)}\right)}^{k}}{\beta }\right)-\frac{\gamma_{i}}{\beta })\right), \end{array}$$

(5b) $$\begin{array}{cc} z_{j}^{k+1} & =\prod_{{\mathrm{PP}}_{d_{c_{j}}}}\left(T_{j}x^{k+1}+\frac{\lambda_{j}^{k}}{\beta }\right), \end{array}$$

(5c) $$\begin{array}{cc} \lambda_{j}^{k+1} & =\lambda_{j}^{k}+\beta \left(T_{j}x^{k+1}-z_{j}^{k+1}\right). \end{array}$$

In the update operation of $x_{i}^{k+1}$, we use ${\left(T\right)}_{i,j}=|d_{v_{i}}|\delta_{i,j}$. For simplicity, we use $z_{j}^{\left(i\right)}$ to represent the *i*-th component of $T_{j}^{T}z_{j}$. Similarly, we use $\lambda_{j}^{\left(i\right)}$ to represent the *i*-th component of $T_{j}^{T}\lambda_{j}$. The $\prod_{\left[0,1\right]}\left(q\right)$ operation represents projecting the vector *q* onto the unit polyhedron. In the update operation of $z_{j}^{k+1}$, the $\prod_{{\mathrm{PP}}_{d_{c_{j}}}}\left(q\right)$ represents the Euclidean projection algorithm of vector q on the test polytope $\prod_{{\mathrm{PP}}_{d_{c_{j}}}}$. But it is not the focus of this paper. In this paper, the projection algorithm proposed in the literature will be directly adopted. For more information on the projection algorithm, please refer to the literature [25].

### 2.2. ADMM-LP Decoding Algorithm with Penalty Term

Although the ADMM-LP decoding algorithm overcomes the problem of error platform in the Message Passing (MP) decoding algorithm, experiments have shown that under low signal-to-noise ratio conditions, the error correction performance of the ADMM-LP algorithm is often lower than that of the MP algorithm under the same conditions. To overcome this drawback, Liu et al. [6] proposed the ADMM algorithm with a penalty term. The core of this algorithm is to add a penalty term to the objective function of Equation (2), making it $\gamma^{T}x+\sum g\left(x_{i}\right)$, and for the ADMM-PD, its mathematical expression is

(6) $$\begin{array}{cc} \min_{x}\; & \gamma^{T}x+\sum_{i}g\left(x_{i}\right),\\ s.\;t.\;T_{j}x & =z_{j},\;z_{j}\in P_{d_{c_{j}}},j\in J. \end{array}$$

For Equation (6), after substituting the ADMM template, the updates of λ and z remain unchanged, except for the update of x, which is transformed from Equation (5a) to Equation (7).

(7) $$x_{i}^{k+1}=\prod_{\left[0,1\right]}\left[\frac{1}{d_{v_{i}}}\left(\sum_{j\in N_{v}\left(i\right)}\left({\left(z_{j}^{\left(i\right)}\right)}^{k}-\frac{{\left(\lambda_{j}^{\left(i\right)}\right)}^{k}}{\beta }\right)\right)-\frac{\gamma_{i}}{\beta }-\frac{1}{\beta }{\left(\nabla_{x}g\right)}_{i}\right)].$$

Regarding the selection of the penalty function g(x), Liu proposed several different penalty functions. For the penalty function g(x)=−α|x−0.5|, it is referred to as the $L_{1}$ penalty function; for $g\left(x\right)={-\alpha ∥x-0.5∥}_{2}^{2}$, it is denoted as ADMM-$L_{2}$. For the penalty function of $L_{2}$, Equation (7) becomes Equation (8).

(8) $$x_{i}=\prod_{\left[0,1\right]}\frac{t_{i}-\frac{\alpha }{\beta }}{d_{v_{i}}-2\frac{\alpha }{\beta }},$$

where $t_{i}$ is defined as $t_{i}=\sum_{j\in N_{v}\left(i\right)}\left(z_{j}^{\left(i\right)}-\frac{1}{\beta }\lambda_{j}^{\left(i\right)}\right)-\frac{1}{\beta }\gamma_{i}$. The parameter α of the penalty function g(x) is a constant greater than 0, and when a is equal to 0, the ADMM-PD decoding algorithm will degenerate into the ADMM-LP decoding algorithm. In the ADMM decoding process, the Over Relaxation (OR) technique can be used to make the algorithm converge to the optimal solution faster. The specific approach of the OR acceleration strategy is to replace $T_{j}x$ in Formulas (5b) and (5c) with

(9) $$\rho T_{j}x+\left(1-\rho \right)z_{j},$$

where ρ is the relaxation coefficient for ρ>1; this technique is called hyper relaxation. The main idea is to perform weighted correction on the latest value of the current iteration and the value of the previous iteration to obtain the best convergence speed.

## 3. S-ADMM Decoder

As we all know, sparsity leads to each variable node being constrained by only a few validation nodes, resulting in weak global coupling. The single dual variable update of the traditional ADMM may not fully coordinate local conflicts, resulting in slow convergence. In addition, in sparse structures, the dependency chains between variable nodes are relatively short, and a single dual variable update may not be able to transmit information in a timely manner, resulting in delayed information transmission. In response to this situation, we adopted a phased strategy to coordinate local conflicts based on the current original variables and historical auxiliary variables. We prioritized the resolving conflicts among some verification nodes based on the differences, and then made further adjustments based on the updated ones. On the basis of the ADMM, some algorithm improvements were made, and the update steps in the ADMM algorithm were changed by introducing a new relaxation factor, so that the selection range of parameters is wider.

Consider the model with $L_{2}$ penalty function

(10) $$\begin{array}{cc} \min_{x}\; & \gamma^{T}x-\alpha {\left(x-0.5\right)}^{2},\\ s.\;t.\;T_{j}x & =z_{j},j\in J. \end{array}$$

Due to the $L_{2}$ penalty function we used, the model becomes a non-convex function. In this paper, we propose an S-ADMM for solving the possibly non-convex optimization problem (10), whose iterative scheme is

(11) $$\left\{\begin{array}{c} x^{k+1}=\underset{x}{\arg\min}\;L_{\beta }\left(x,z^{k},\lambda_{j}^{k}\right),\\ \lambda_{j}^{k+\frac{1}{2}}=\lambda_{j}^{k}+\eta \beta \left(T_{j}x^{k+1}-z_{j}^{k}\right),\\ z_{j}^{k+1}=\underset{z}{\arg\min}\;L_{\beta }\left(x^{k+1},z_{j},\lambda_{j}^{k+\frac{1}{2}}\right),\\ \lambda_{j}^{k+1}=\lambda_{j}^{k+\frac{1}{2}}+\beta \left(T_{j}x^{k+1}-z_{j}^{k+1}\right). \end{array}\right.$$

In the S-ADMM iterative format (11), we added an update to the Lagrange multiplier (i.e., $\lambda^{k+\frac{1}{2}}$). At the same time, in order to improve the numerical performance of the algorithm, we introduced a parameter η for this term. For different codes in the simulation, the value of η makes the iteration more representative. Here η∈(−1,1). Note that for η=0, the iterative scheme (11) reduces to the classic ADMM (4). As we will demonstrate, introducing an additional relaxation factor into the S-ADMM scheme (11) plays a crucial role in ensuring that the sequence generated by the algorithm exhibits a strictly contractive behavior relative to the solution set of (10). This contraction property allows us to establish rigorous worst-case convergence rates for the S-ADMM method. Importantly, this analysis can be conducted without the need for further assumptions or modifications to the underlying model (10), highlighting the robustness of the scheme’s convergence guarantees.

For model (10), the specific iteration rules for each variable are as follows:

(12) $$\begin{array}{cc} x^{k+1} & =\underset{x}{\arg\min}\;L_{\beta }\left(x,z_{j}^{k},\lambda_{j}^{k}\right)\\ \; & =\prod_{\left[0,1\right]}\frac{\sum_{j\in N_{v}\left(i\right)}\left({\left(z_{j}^{\left(i\right)}\right)}^{k}-\frac{1}{\beta }{\left(\lambda_{j}^{\left(i\right)}\right)}^{k}\right)-\frac{\gamma_{i}}{\beta }-\frac{\alpha }{\beta }}{d_{v_{i}}-2\frac{\alpha }{\beta }}, \end{array}$$

(13) $$\lambda_{j}^{k+\frac{1}{2}}=\lambda_{j}^{k}+\eta \beta \left(T_{j}x^{k+1}-z_{j}^{k}\right),$$

(14) $$\begin{array}{cc} z_{j}^{k+1} & =\underset{z}{\arg\min}\;L_{\beta }\left(x^{k+1},z,\lambda_{j}^{k+\frac{1}{2}}\right)\\ \; & =\prod_{P_{d_{c_{j}}}}\left(T_{j}x^{k+1}+\frac{\lambda_{j}^{k+\frac{1}{2}}}{\beta }\right), \end{array}$$

(15) $$\lambda_{j}^{k+1}=\lambda_{j}^{k+\frac{1}{2}}+\beta \left(T_{j}x^{k+1}-z_{j}^{k+1}\right).$$

Algorithm 1 provides the specific process of the S-ADMM decoding algorithm. From the specific implementation of the algorithm, it can be seen that the entire decoding algorithm is still an iterative architecture, similar to the BP decoding algorithm, where data are still passed back and forth between variable nodes and check nodes for iterative operations.
**Algorithm 1** Decoding Algorithm Based on S-ADMM.1.Construct a non-negative LLR vector; γ is based on the received vector *w*.2.Construct the selection matrix; $T_{j}$ corresponds to each row of the check matrix.3.Initialization, $\lambda^{0}$ is a full 0 vector and $z^{0}$ is a full 0.5 vector.4.**repeat**5.iter← iter+16.    **for** all i∈I do7.       $x^{k+1}=\prod_{\left[0,1\right]}\frac{\sum_{j\in N_{v}\left(i\right)}\left({\left(z_{j}^{\left(i\right)}\right)}^{k}-\frac{1}{\beta }{\left(\lambda_{j}^{\left(i\right)}\right)}^{k}\right)-\frac{\gamma_{i}}{\beta }-\frac{\alpha }{\beta }}{d_{v_{i}}-2\frac{\alpha }{\beta }}$8.    **end** for9.    **for** all j∈J do10.        $\lambda_{j}^{k+\frac{1}{2}}=\lambda_{j}^{k}+\eta \beta \left(T_{j}x^{k+1}-z_{j}^{k}\right)$11.        $z_{j}^{k+1}=\prod_{P_{d_{c_{j}}}}\left(T_{j}x^{k+1}+\frac{\lambda_{j}^{k+\frac{1}{2}}}{\beta }\right)$12.        $\lambda_{j}^{k+1}=\lambda_{j}^{k+\frac{1}{2}}+\beta \left(T_{j}x^{k+1}-z_{j}^{k+1}\right)$13.    **end** for14.**until** $\sum_{j}{∥T_{j}x^{k}-z_{j}^{k}∥}_{2}^{2}<\epsilon^{2}$ or iter $\geq I_{max}$15.**return** x

In each iteration of the ADMM algorithm, the computational complexity for updating the variable *x* is O(n). Similarly, the update of the auxiliary variable z involves a complexity of O(m). The update of the Lagrange multiplier λ also incurs a complexity of O(m). When we incorporate the S-ADMM scheme, an additional step is introduced where the intermediate variable λ is updated. However, the complexity of this update remains O(m), which is consistent with the complexity of updating z and λ in the ADMM scheme. In Table 1, we provide the number of operations and complexity for each step of the S-ADMM algorithm, where $D_{v}$ represents the maximum column weight in the matrix *H* and $D_{c}$ represents the maximum row weight in the matrix *H*. Thus, the overall complexity of the S-ADMM method is dominated by the updates of x, z, and λ, with the intermediate update of λ not significantly affecting the total computational cost. In addition, in Table 2, we list the updates of variables, average iteration times, and complexity of shrinkage properties for the S-ADMM algorithm and A-ADMM-$L_{2}$ algorithm.

In the following analysis, we turn our attention to comparing the average number of iterations required for the algorithm to converge to the correct codeword, providing insight into the efficiency of the S-ADMM scheme in practical applications.

## 4. Algorithm and Contraction Analysis

### 4.1. Variational Reformulation of Equation (10)

The Lagrangian function of the model (10) is as follows:

(16) $$L\left(x,z,\lambda \right)=\gamma^{T}x-\alpha {\left(x-0.5\right)}^{2}+\sum_{j}\lambda_{j}^{T}\left(T_{j}x-z_{j}\right)+\frac{\beta }{2}\sum_{j}{∥T_{j}x-z_{j}∥}_{2}^{2}.$$

To solve problem (10), we need to find a solution $y^{⋆}=\left(x^{⋆},z^{⋆},\lambda^{⋆}\right)$ that [26]

(17) $$\phi \left(u\right)-\phi \left(u^{⋆}\right)+{\left(y-y^{⋆}\right)}^{T}F\left(y^{⋆}\right)\geq 0.$$

Suppose

$$\phi_{1}\left(x\right)=\gamma^{T}x-\alpha {\left(x-0.5\right)}^{2},\phi_{2}\left(z\right)=0\cdot z,\phi \left(u\right)=\phi_{1}\left(x\right)+\phi_{2}\left(z\right),$$

where

(18) $$u=\left(\begin{array}{c} x\\ z \end{array}\right),y=\left(\begin{array}{c} x\\ z\\ \lambda \end{array}\right),F\left(y\right)=\left(\begin{array}{c} \sum_{j}T_{j}^{T}\lambda_{j}\\ -\sum_{j}\lambda_{j}\\ -(T_{j}x-z_{j}) \end{array}\right).$$

**Theorem** **1.** *If $\Omega^{⋆}$ represent the solution set of the optimization problem V(Ω,F,ϕ), then*

(19) $$\Omega^{*}=\underset{y\in \Omega }{⋂}\left\{\tilde{y}\in \Omega :\phi \left(u\right)-\phi \left(\tilde{u}\right)+{\left(y-\tilde{y}\right)}^{T}F\left(y\right)\geq 0\right\}.$$

Its proof can be seen in [27]. Theorem 1 states that if $\tilde{y}\in \Omega$ satisfies

(20) $$\underset{y\in D}{\sup}\left\{\phi \left(\tilde{u}\right)-\phi \left(u\right)+{\left(\tilde{y}-y\right)}^{T}F\left(y\right)\right\}\leq \epsilon ,$$

with D∈Ω, then $\tilde{y}$ is an approximate solution to the set V(Ω,F,ϕ).

### 4.2. Some Notation

For the S-ADMM, when solving for variable $x^{k+1}$, it is related to $z^{k}$ and $\lambda^{k}$ in (12), so we define a set $s^{k}$ such that $s^{k}=\left(z^{k},\lambda^{k}\right)$,

(21) $$S^{⋆}:=\left\{s^{⋆}=\left(z^{⋆},\lambda^{⋆}\right)|y^{⋆}=\left(x^{⋆},z^{⋆},\lambda^{⋆}\right)\right\}.$$

For convenience, we define the following matrices.

(22) $$M=\left(\begin{array}{cc} I_{d_{c_{j}}} & 0\\ -\beta I_{d_{c_{j}}} & \left(\eta +1\right)I_{d_{c_{j}}} \end{array}\right),$$

and

(23) $$Q_{O}=\left(\begin{array}{cc} 0 & 0\\ \beta I_{d_{c_{j}}} & -\eta I_{d_{c_{j}}}\\ -I_{d_{c_{j}}} & \frac{1}{\beta }I_{d_{c_{j}}} \end{array}\right),$$

We further define

(24) $$Q=\left(\begin{array}{cc} \beta I_{d_{c_{j}}} & -\eta I_{d_{c_{j}}}\\ -I_{d_{c_{j}}} & \frac{1}{\beta }I_{d_{c_{j}}} \end{array}\right),$$

(25) $$A=\frac{1}{\eta +1}\left(\begin{array}{cc} \beta I_{d_{c_{j}}} & -\eta I_{d_{c_{j}}}\\ -\eta I_{d_{c_{j}}} & \frac{1}{\beta }I_{d_{c_{j}}} \end{array}\right).$$

Notice that

(26) $$A=\frac{1}{\eta +1}{\left(\begin{array}{cc} \sqrt{\beta }I_{d_{c_{j}}} & 0\\ 0 & \sqrt{\frac{1}{\beta }}I_{d_{c_{j}}} \end{array}\right)}^{T}\left(\begin{array}{cc} I_{d_{c_{j}}} & -\eta I_{d_{c_{j}}}\\ -\eta I_{d_{c_{j}}} & I_{d_{c_{j}}} \end{array}\right)\left(\begin{array}{cc} \sqrt{\beta }I_{d_{c_{j}}} & 0\\ 0 & \sqrt{\frac{1}{\beta }}I_{d_{c_{j}}} \end{array}\right),$$

and the matrix

$$\left(\begin{array}{cc} 1 & -\eta \\ -\eta & 1 \end{array}\right)$$

is positive definite for η∈(−1,1) and positive semidefinite for η=1.

The convergence rate analysis of the iterative process defined in Equation (11) relies heavily on this contraction property. By examining the contraction behavior of the sequence, we can establish the conditions under which the sequence $s^{k}$ converges to the optimal solution and derive the associated convergence rate. This contraction analysis for Equation (11) is crucial because it provides a rigorous mathematical framework to quantify how quickly the iterates $s^{k}$ approach the optimal set $S^{*}$. More specifically, we investigate the rate of decay of the distance between successive iterates and the optimal solution set, ensuring that each step of the S-ADMM method brings the sequence closer to convergence. This analysis will form the foundation for deriving the convergence rate, as it characterizes the speed at which the algorithm converges under appropriate conditions. We focus here solely on the contraction properties of Equation (11), as these properties directly influence the overall efficiency and convergence speed of the S-ADMM scheme.

### 4.3. Contraction Analysis

In this section, we establish the contraction property of the sequence $s^{k}$, generated by (11), with respect to the set $S^{⋆}$.

Here we define a set ${\tilde{y}}^{k}$ as

(27) $${\tilde{y}}^{k}=\left(\begin{array}{c} {\tilde{x}}^{k}\\ {\tilde{z}}_{j}^{k}\\ {\tilde{\lambda }}_{j}^{k} \end{array}\right)=\left(\begin{array}{c} x^{k+1}\\ z_{j}^{k+1}\\ \lambda_{j}^{k}+\beta \left(T_{j}x^{k+1}-z_{j}^{k}\right) \end{array}\right),$$

where $\left(x^{k+1},z_{j}^{k+1}\right)$ is generated by (11). Note that with the notation of ${\tilde{y}}^{k}$, we immediately have

(28) $$x^{k+1}={\tilde{x}}^{k},z_{j}^{k+1}={\tilde{z}}_{j}^{k},\lambda_{j}^{k+\frac{1}{2}}=\lambda_{j}^{k}-\eta \left(\lambda_{j}^{k}-{\tilde{\lambda }}_{j}^{k}\right).$$

Then, based on (11) and (28), we immediately get

(29) $$\begin{array}{cc} \lambda_{j}^{k+1} & =\lambda_{j}^{k+\frac{1}{2}}+\beta \left(T_{j}{\tilde{x}}^{k}-{\tilde{z}}_{j}^{k}\right)\\ \; & =\lambda_{j}^{k}-\eta \left(\lambda_{j}^{k}-{\tilde{\lambda }}_{j}^{k}\right)+\left[\beta \left(T_{j}{\tilde{x}}^{k}-z_{j}^{k}\right)+\beta \left(z_{j}^{k}-{\tilde{z}}_{j}^{k}\right)\right]\\ \; & =\lambda_{j}^{k}-\eta \left(\lambda_{j}^{k}-{\tilde{\lambda }}_{j}^{k}\right)+\left[\left(-\lambda_{j}^{k}+{\tilde{\lambda }}_{j}^{k}\right)+\beta \left(z_{j}^{k}-{\tilde{z}}_{j}^{k}\right)\right]\\ \; & =\lambda_{j}^{k}-\left(\eta +1\right)\left(\lambda_{j}^{k}-{\tilde{\lambda }}_{j}^{k}\right)+\beta \left(z_{j}^{k}-{\tilde{z}}_{j}^{k}\right). \end{array}$$

Furthermore, together with $z^{k+1}={\tilde{z}}^{k}$, we have the relationship

(30) $$\left(\begin{array}{c} z_{j}^{k+1}\\ \lambda_{j}^{k+1} \end{array}\right)=\left(\begin{array}{c} z_{j}^{k}\\ \lambda_{j}^{k} \end{array}\right)-\left(\begin{array}{cc} I_{d_{c_{j}}} & 0\\ -\beta I_{d_{c_{j}}} & \left(\eta +1\right)I_{d_{c_{j}}} \end{array}\right)\left(\begin{array}{c} z_{j}^{k}-{\tilde{z}}_{j}^{k}\\ \lambda_{j}^{k}-{\tilde{\lambda }}_{j}^{k} \end{array}\right),$$

which can be rewritten compactly as

(31) $$s^{k+1}=s^{k}-M\left(s^{k}-{\tilde{s}}^{k}\right).$$

Next, we will explain that the algorithm (11) is convergent.

**Lemma** **1.** *For given $s^{k}$, let $y^{k+1}$ be generated by the S-ADMM scheme. Then,*

(32) $$\phi \left(u\right)-\phi \left({\tilde{u}}^{k}\right)+{\left(y-{\tilde{y}}^{k}\right)}^{T}F\left({\tilde{y}}^{k}\right)\geq {\left(s-{\tilde{s}}^{k}\right)}^{T}Q\left(s^{k}-{\tilde{s}}^{k}\right),$$

*and ${\tilde{y}}^{k}$ is a solution of V(Ω,F,ϕ) if $∥s^{k}-s^{k+1}{∥}_{A}^{2}=0$.*

**Proof.**  Since $x^{k+1}={\tilde{x}}^{k}$, for the update of x in Equation (11), we have

(33) $$\phi \left(x\right)-\phi \left({\tilde{x}}^{k}\right)+{\left(x-{\tilde{x}}^{k}\right)}^{T}\left\{\sum_{j}T_{j}^{T}\left[\lambda_{j}^{k}+\beta \left(T_{j}{\tilde{x}}^{k}-z_{j}^{k}\right)\right]\right\}\geq 0.$$

According to the definition (27), we have

(34) $${\tilde{\lambda }}_{j}^{k}=\lambda_{j}^{k}+\beta \left(T_{j}x^{k+1}-z_{j}^{k}\right).$$

Using (34), the inequality (33) will become

(35) $$\phi \left(x\right)-\phi \left({\tilde{x}}^{k}\right)+{\left(x-{\tilde{x}}^{k}\right)}^{T}\left(\sum_{j}T_{j}^{T}{\tilde{\lambda }}_{j}^{k}\right)\geq 0.$$

Similarly, for the update of z in Equation (11), we have

(36) $${\left(z-{\tilde{z}}^{k}\right)}^{T}\left(-\sum_{j}\left[\lambda_{j}^{k}-\eta \left(\lambda_{j}^{k}-{\tilde{\lambda }}_{j}^{k}\right)\right]-\beta \sum_{j}\left(T_{j}{\tilde{x}}^{k}-{\tilde{z}}^{k}\right)\right)\geq 0.$$

Reusing (34),we have

(37) $$\begin{array}{c} \lambda_{j}^{k}-\eta \left(\lambda_{j}^{k}-{\tilde{\lambda }}_{j}^{k}\right)+\beta \left(T_{j}{\tilde{x}}^{k}-{\tilde{z}}^{k}\right)\\ =\lambda_{j}^{k}+\eta \left({\tilde{\lambda }}_{j}^{k}-\lambda_{j}^{k}\right)-\beta \left({\tilde{z}}_{j}^{k}-z_{j}^{k}\right)+\left({\tilde{\lambda }}_{j}^{k}-\lambda_{j}^{k}\right)\\ ={\tilde{\lambda }}_{j}^{k}+\eta \left({\tilde{\lambda }}_{j}^{k}-\lambda_{j}^{k}\right)-\beta \left({\tilde{z}}_{j}^{k}-z_{j}^{k}\right). \end{array}$$

Consequently, it follows from (36) that

(38) $${\left(z-{\tilde{z}}^{k}\right)}^{T}\left\{\sum_{j}\left[-{\tilde{\lambda }}_{j}^{k}-\eta \left({\tilde{\lambda }}_{j}^{k}-\lambda_{j}^{k}\right)+\beta \left({\tilde{z}}_{j}^{k}-z_{j}^{k}\right)\right]\right\}\geq 0.$$

Meanwhile,

(39) $$\left(T_{j}{\tilde{x}}^{k}-{\tilde{z}}_{j}^{k}\right)+\left({\tilde{z}}_{j}^{k}-z_{j}^{k}\right)-\frac{1}{\beta }\left({\tilde{\lambda }}^{k}-\lambda^{k}\right)=0.$$

Combining (35), (38), and (39), we get

(40) $$\phi \left(u\right)-\phi \left({\tilde{u}}^{k}\right)+{\left(\begin{array}{c} x-{\tilde{x}}^{k}\\ z-{\tilde{z}}^{k}\\ \lambda -{\tilde{\lambda }}^{k} \end{array}\right)}^{T}\left(\left(\begin{array}{c} \sum_{j}T_{j}^{T}{\tilde{\lambda }}_{j}^{k}\\ -\sum_{j}{\tilde{\lambda }}_{j}^{k}\\ -(T_{j}{\tilde{x}}^{k}-{\tilde{z}}_{j}^{k}) \end{array}\right)+\left(\begin{array}{c} 0\\ \beta \left({\tilde{z}}^{k}-z^{k}\right)-\eta \left({\tilde{\lambda }}^{k}-\lambda^{k}\right)\\ -\left({\tilde{z}}^{k}-z^{k}\right)+\frac{1}{\beta }\left({\tilde{\lambda }}^{k}-\lambda^{k}\right) \end{array}\right)\right)\geq 0.$$

Equation (32) represents only one form of the above inequality. At last, we get

$$\phi \left(u\right)-\phi \left({\tilde{u}}^{k}\right)+{\left(y-{\tilde{y}}^{k}\right)}^{T}F\left({\tilde{y}}^{k}\right)\geq {\left(s-{\tilde{s}}^{k}\right)}^{T}Q\left(s^{k}-{\tilde{s}}^{k}\right).$$

Based on this, from Q=AM and $s^{k+1}=s^{k}-M\left(s^{k}-{\tilde{s}}^{k}\right)$, we obtain

(41) $${\left(s-{\tilde{s}}^{k}\right)}^{T}Q\left(s^{k}-{\tilde{s}}^{k}\right)={\left(s-{\tilde{s}}^{k}\right)}^{T}A\left(s^{k}-s^{k+1}\right).$$

Apply the above equation to (32):

(42) $$\phi \left(u\right)-\phi \left({\tilde{u}}^{k}\right)+{\left(y-{\tilde{y}}^{k}\right)}^{T}F\left({\tilde{y}}^{k}\right)\geq {\left(s-{\tilde{s}}^{k}\right)}^{T}A\left(s^{k}-s^{k+1}\right).$$

Note that A is a symmetric positive definite matrix. From (32) and $∥s^{k}-s^{k+1}{∥}_{A}^{2}=0$, it can be inferred that $A(s^{k}-s^{k+1})=0$, and thus $\phi \left(u\right)-\phi \left(\tilde{u}\right)+{\left(y-\tilde{y}\right)}^{T}F\left({\tilde{y}}^{k}\right)\geq 0$. According to (19), ${\tilde{y}}^{k}$ is the solution. The proof is completed. □

## 5. Simulation Result

### 5.1. Parameter Selection

In this section, we selected several different codes for experimentation and compared the reliability and complexity of the designed S-ADMM decoding algorithm with the ADMM-$L_{2}$ algorithm and A-ADMM-$L_{2}$ algorithm. Among them, the A-ADMM-$L_{2}$ represents the optimization-based ADMM-$L_{2}$ algorithm, which uses the differential evolution (DE) algorithm. They are, respectively, referred to as $C_{1}$ (WIMAX (576,288), rate $\frac{1}{2}$), $C_{2}$ (802.11n (648,216), rate $\frac{3}{4}$), $C_{3}$((1152,288), rate $\frac{3}{4}$), and three types of 5G LDPC codes with the information length 320 but different rates. In the 5G standard, the performance requirements for the design of 5G LDPC codes are higher. In Table 3, we list the parameter information for six types of codes.

Since the codes are all irregular codes, we assign a different parameter to each variable node when doing the simulation, i.e., parameter α. For code $C_{1}$, the parameters for ADMM-$L_{2}$ are directly selected from the parameters provided in reference [20]. In this paper, μ=4.0 and α=2.2. Here, we provide the FER corresponding to different parameters of $C_{1}$, $C_{2}$, and $C_{4}$ codes, and similar methods are also used for other codes. The SNR of code $C_{1}$ is 2.0 dB, the SNR of code $C_{2}$ is 2.8 dB, and the SNR of code $C_{4}$ is 2.8 dB. The FER performances of the three codes are evaluated over the additive white Gaussian noise channel (AWGNC) using binary phase-shift keying (BPSK) modulation. The corresponding results for each code are depicted in Table 4, Table 5 and Table 6, respectively. These tables illustrate the impact of different values of parameters on the error performance, providing a comparative analysis of the effectiveness of each code in mitigating frame errors under the given channel conditions.

**Figure 1:**
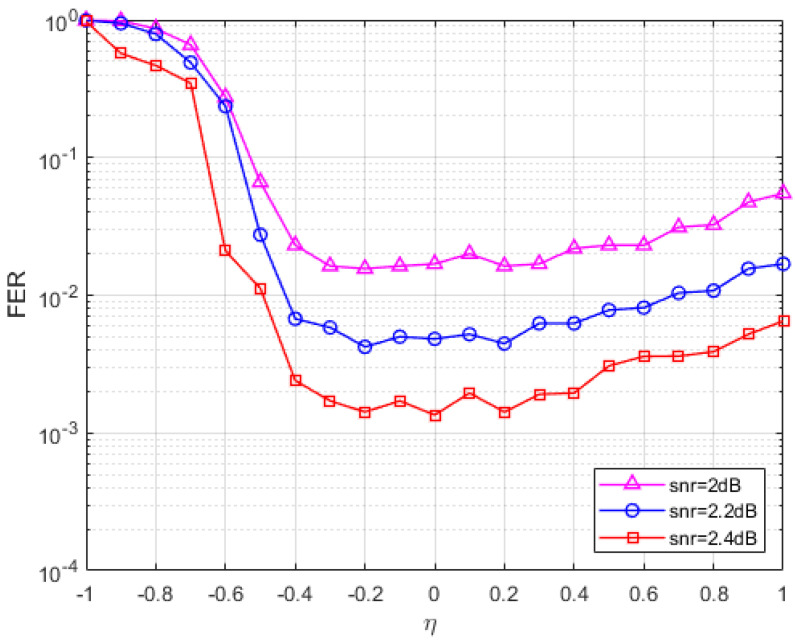
The FER of $C_{1}$ for the S-ADMM decoder under different η.

**Figure 2:**
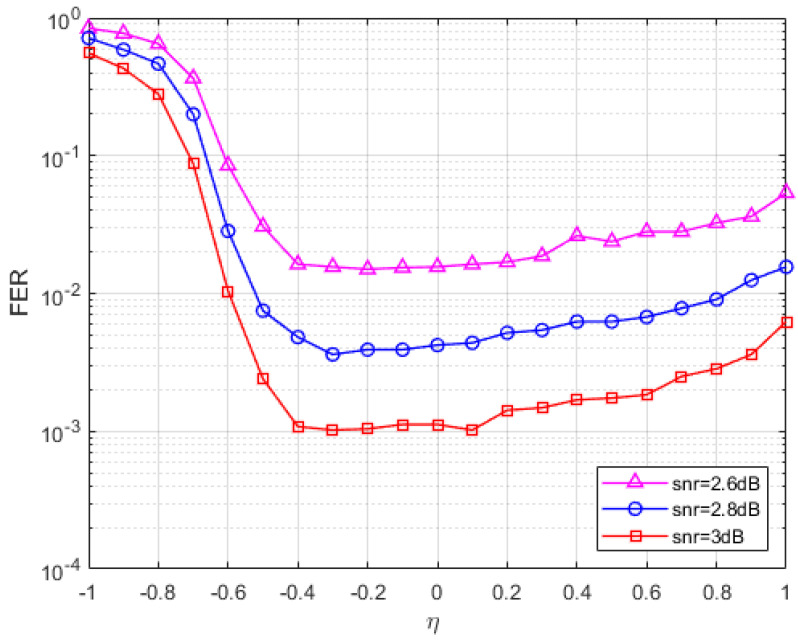
The FER of $C_{2}$ for the S-ADMM decoder under different η.

**Figure 3:**
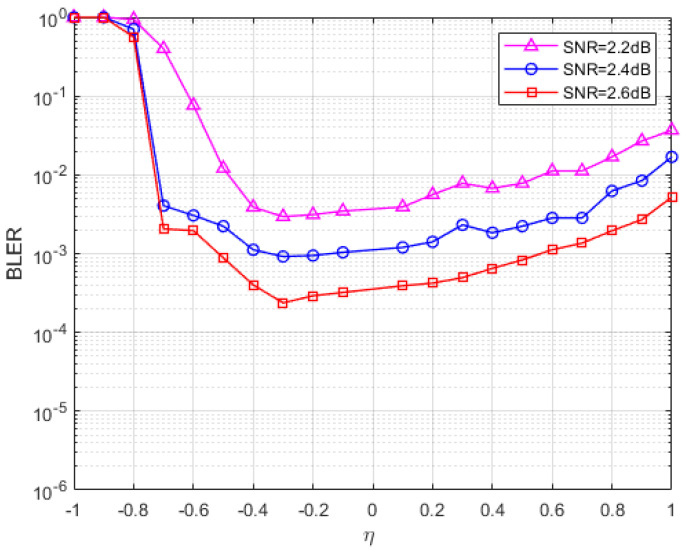
The block error rate (BLER) of $C_{4}$ for the S-ADMM decoder under different η.

In Figure 1, the SNR and penalty parameters are fixed, and the FER of the parameters at different values is plotted. At least 200 errors were collected for each data point at three fixed SNRs (SNR = 2 dB, 2.2 dB, 2.4 dB). The maximum number of iterations in Figure 1 is set to 1000 times, and it can be seen from the figure that when the value is less than −0.3, the FER curve of the S-ADMM decoding algorithm decreases with the increase in the value, and when the parameter value is between −0.3 and 0.4, the FER reaches a lower peak, and when the value is greater than 0.3, the FER curve has a tendency to rise and increase after that. It can be found that when the parameter value is between −0.3 and 0.2, the S-ADMM decoder has better error correction performance. So, we choose a relatively stable value of −0.15. Similarly, for the $C_{2}$ code in Figure 2, we find a stable value of −0.3 and a similar one for $C_{4}$ in Figure 3. In the following performance analysis, we will conduct experiments using the analyzed optimal parameters.

### 5.2. Performance Analysis

**Figure 4:**
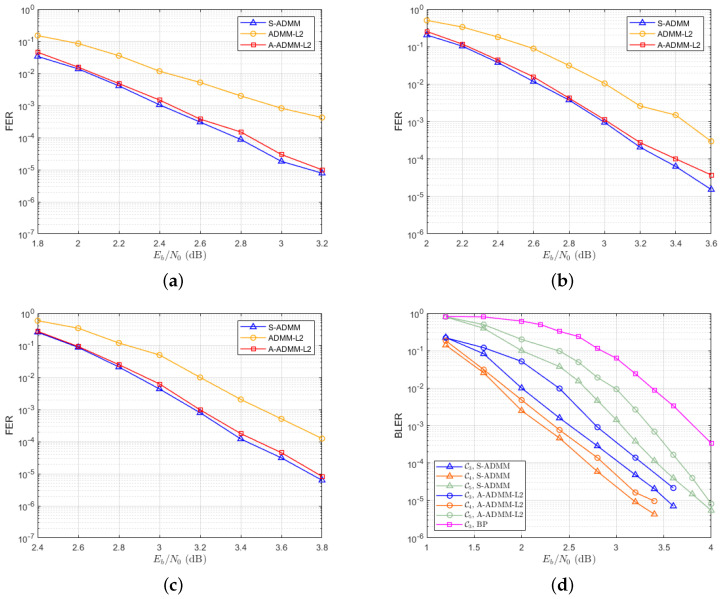
Decoding performance corresponding to six types of codes. They are listed as follows: (**a**) The FER of the S-ADMM, ADMM-$L_{2}$, and A-ADMM-$L_{2}$ decoders of $C_{1}$ code. (**b**) The FER of the S-ADMM, ADMM-$L_{2}$, and A-ADMM-$L_{2}$ decoders of $C_{2}$ code. (**c**) The FER of the S-ADMM, ADMM-$L_{2}$, and A-ADMM-$L_{2}$ decoders of $C_{3}$ code. (**d**) The BLER of the S-ADMM, BP, and A-ADMM-$L_{2}$ decoders of three types of 5G codes.

Figure 4 compares the FER performance of several decoding algorithms for different codes. From Figure 4a, it can be seen that for code $C_{1}$, compared to the ordinary ADMM-$L_{2}$ and A-ADMM-$L_{2}$ decoding algorithms, the S-ADMM decoding algorithm can achieve a decoding performance improvement of nearly 0.1 dB. For example, at a frame error rate of $10^{-3}$, the SNR corresponding to the ADMM-$L_{2}$ decoding algorithms is approximately 3 dB. The SNR corresponding to the A-ADMM-$L_{2}$ decoding algorithms is approximately 2.5 dB. The corresponding SNR of the S-ADMM decoding algorithm is about 2.4 dB. Similarly, from Figure 4b, it can be seen that for code $C_{2}$, compared to other decoding algorithms, the S-ADMM decoding algorithm still has better decoding performance, which is also true for the high-rate $C_{3}$ code in Figure 4c. On the other hand, for the 5G LDPC codes in Figure 4d, compared with S-ADMM and BP decoding algorithms, under high SNR conditions, it can be clearly seen that the BP algorithm has the problem of error platform, while the S-ADMM decoding algorithm can still maintain good decoding performance. At a frame error rate of $10^{-2}$, the S-ADMM decoding algorithm can achieve a decoding performance improvement of nearly 1.5 dB more than BP.

### 5.3. Average Number of Decoding Iterations

**Figure 5:**
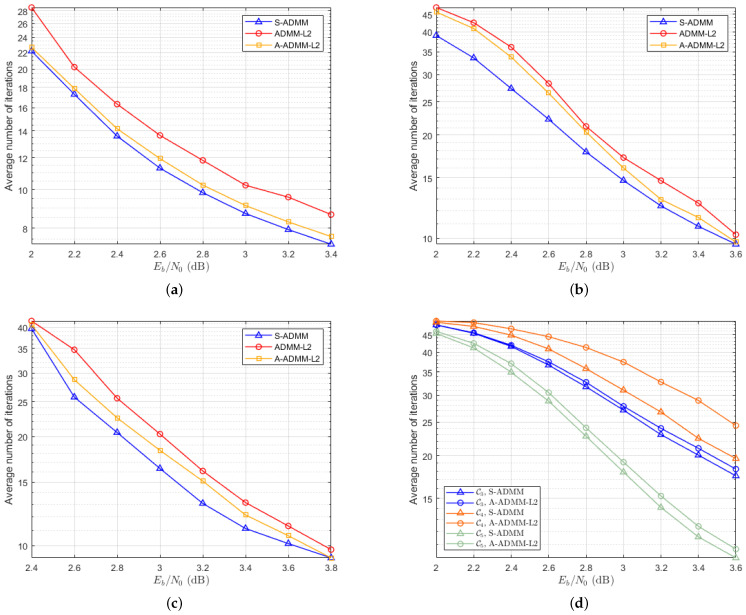
The average number of decoding iterations corresponding to six types of codes. They are listed as follows: (**a**) The average number of iterations for code $C_{1}$. (**b**) The average number of iterations for code $C_{2}$. (**c**) The average number of iterations for code $C_{3}$. (**d**) The average number of iterations of 5G LDPC codes.

Figure 5 illustrates the average number of decoding iterations required by the ADMM-$L_{2}$, the A-ADMM-$L_{2}$, and the S-ADMM decoding algorithms for different codes. Specifically, when the SNR is 2.8 dB, the ADMM-$L_{2}$ requires an average of 11.8 and 22.6 iterations, respectively, for codes $C_{1}$ in Figure 5a and $C_{2}$ in Figure 5b. For the A-ADMM-$L_{2}$, the average number of iterations decreases to 10.4 and 21.4, respectively. However, the S-ADMM decoding algorithm achieves even faster convergence, with average iteration counts of 9.2 and 18.2 for the same codes. This represents a reduction of approximately 22.1% and 19.5%, respectively, in the average number of decoding iterations compared to the ADMM-$L_{2}$. For high-rate $C_{3}$, the S-ADMM algorithm also reduces the average number of decoding iterations in Figure 5c. Figure 5d further demonstrates the comparative performance of the S-ADMM and A-ADMM-$L_{2}$ decoding algorithms for 5G LDPC codes. It is clear from the results that the S-ADMM decoding algorithm significantly accelerates convergence relative to A-ADMM-$L_{2}$ decoding, further emphasizing its efficiency in iterative decoding scenarios. These findings highlight the superior performance of the S-ADMM decoding approach, not only in terms of decoding speed but also in its ability to reduce computational complexity.

## 6. Conclusions

In this paper, we addressed the issue of slow convergence speed in the ADMM when applied to LP models with penalty functions. Specifically, we proposed an enhanced ADMM decoding algorithm, where we introduced an intermediate update of the multipliers within the ADMM iteration scheme to accelerate convergence and improve performance.

Based on the proof framework of variational inequalities, we studied the contraction properties of algorithms, analyzed the complexity and optimal parameter values of algorithms, and verified the feasibility of the algorithms. Additionally, we presented a brief performance analysis of LDPC codes over the AWGNC, highlighting the advantages of our approach in comparison to existing methods.

Furthermore, we described the detailed algorithmic process for implementing the S-ADMM algorithm, outlining its key steps and computational considerations. Numerical results demonstrated that the S-ADMM algorithm significantly outperforms traditional ADMM penalized decoders. These findings suggested that our approach offers a promising solution for improving the efficiency of ADMM decoding algorithms, particularly in the context of LP models with penalty functions.

The symmetric ADMM algorithm presented in this paper provides an efficient and stable new method for LDPC code decoding, but its practical deployment still requires continuous breakthroughs in theoretical completeness, hardware optimization, and cross technology integration. Future research can focus on the full chain innovation of theory algorithm system scenario, promoting the deep application of the ADMM framework in next-generation communication systems.

## Figures and Tables

**Table 1:** The number of operations and the complexity of the algorithm execution.

Step	Number of Operations	Complexity
1	*n*	-
2	*m*	-
3	$d_{c_{j}}$	-
6	$d_{v_{i}}$	$O(n\cdot D_{v})$
9	$d_{c_{j}}$	$O(m\cdot D_{c})$
10	$d_{c_{j}}$	$O(m\cdot D_{c})$
11	$d_{c_{j}}$	$O(m\cdot D_{c})$

**Table 2:** Comparison of complexity between S-ADMM algorithm and A-ADMM-$L_{2}$ algorithm.

Algorithm	S-ADMM	A-ADMM-$l_{2}$	Comparison of Complexity
Update of *x*	$O(n\cdot D_{v})$	$O(n\cdot D_{v})$	Same, both have linear complexity.
Update of *z*	$O(m\cdot D_{c})$	$O(m\cdot D_{c})$	The projection algorithms used are all from reference [25], with the same complexity.
Update of λ	2×O(m)	1×O(m)	S-ADMM has an additional dual update, but it is only a linear operation and the actual cost can be ignored.
Average number of iterations	Less (Due to the two-stage balance adjustment, oscillation is suppressed.)	Many, due to the single update direction being prone to oscillation.	Symmetrical design balances the adjustment direction of dual variables and accelerates convergence.
Convergence stability	Theorem 1 guarantees strict monotonic convergence.	Relying on the convergence of the traditional ADMM may lead to local oscillations.	S-ADMM suppresses oscillations and reduces ineffective iterations.

**Table 3:** Six types of LDPC codes.

Code	Symbol	Rate	Column Redistribution
(576,288)	$C_{1}$	$\frac{1}{2}$	{2,3,6}
(648,216)	$C_{2}$	$\frac{3}{4}$	{2,3,4,6,8}
(1,152,288)	$C_{3}$	$\frac{3}{4}$	{2,3,6}
320	$C_{4}$	$\frac{1}{2}$	{1,2,3,4,5,7,8}
320	$C_{5}$	$\frac{2}{5}$	{1,2,3,4,5,7,8,10,11}
320	$C_{6}$	$\frac{2}{3}$	{1,2,3,4,5}

**Table 4:** $C_{1}$ code improves the parameter values of the penalty function and the S-ADMM decoding algorithm.

Decoding Algorithm	A-ADMM-$L_{2}$	S-ADMM
$\alpha_{1}$	5.11368	0.00001
$\alpha_{2}$	1.00586	1.90024
$\alpha_{3}$	0.30138	5.42336
β	3.29866	4.15607

**Table 5:** $C_{2}$ code improves the parameter values of the penalty function and the S-ADMM decoding algorithm.

Decoding Algorithm	A-ADMM-$L_{2}$	S-ADMM
$\alpha_{1}$	0.12794	0.06876
$\alpha_{2}$	0.76466	1.13234
$\alpha_{3}$	1.90017	1.60122
$\alpha_{4}$	2.94728	5.29668
$\alpha_{5}$	6.16048	6.44272
β	3.60323	3.17501

**Table 6:** $C_{4}$ code improves the parameter values of the penalty function and the S-ADMM decoding algorithm.

Decoding Algorithm	A-ADMM-$L_{2}$	S-ADMM
$\alpha_{1}$	0.00001	0.00290
$\alpha_{2}$	0.00001	0.00001
$\alpha_{3}$	1.45949	1.53340
$\alpha_{4}$	0.00001	4.09405
$\alpha_{5}$	2.56477	2.17151
$\alpha_{6}$	6.49895	3.05173
$\alpha_{7}$	10.14689	7.49879
β	9.48708	5.93976

## Data Availability

Data are contained within the article.
